# Corynebacterium diphtheriae Septic Arthritis: A Case Report and Literature Review

**DOI:** 10.7759/cureus.91884

**Published:** 2025-09-09

**Authors:** Tee Hang Chia, E Shan Heng

**Affiliations:** 1 Internal Medicine, Queen Elizabeth University Hospital, Glasgow, GBR

**Keywords:** diphtheria, diphtheria antitoxin, elbow swelling, septic arthritis elbow, vaccine, vaccine-preventable disease

## Abstract

Classical respiratory diphtheria, characterised by membranous pharyngitis caused by the gram-positive bacterium *Corynebacterium diphtheriae*, has become increasingly rare due to widespread vaccination. However, invasive manifestations such as septic arthritis and bacteraemia, while still exceptionally rare, are being reported more frequently, particularly in unvaccinated or partially vaccinated individuals and in cases involving non-toxigenic strains.

This case describes a 19-year-old man who presented with various systemic symptoms along with left elbow swelling, initially thought to be reactive arthritis. He was later diagnosed with septic arthritis and bacteraemia secondary to a non-toxigenic strain of *C. diphtheriae* based on the culture result. Treatment involves antimicrobial therapy and diphtheria antitoxin. This case highlights the importance of considering unusual manifestations of diphtheria and the role of antibiotics as well as diphtheria antitoxin in managing toxigenic and non-toxigenic strains of *C. diphtheriae*.

## Introduction

First described by Loeffler [1] in 1884, diphtheria is an infectious disease caused by *Corynebacterium diphtheriae*, typically transmitted through respiratory droplets or direct contact with cutaneous lesions. The disease classically resulted from exotoxin production, leading to symptoms like sore throat, rhinorrhoea, neck swelling, and the development of a pseudomembrane over the tonsils, alongside systemic manifestations such as fever and malaise. Diphtheria was once a leading cause of mortality, particularly among children, until the introduction of diphtheria antitoxin in the 1940s, which drastically reduced cases from over 61,000 in 1940 to just around 40 by 1957 [2].

However, in recent years, there has been a rise in non-toxigenic *C. diphtheriae* infections, especially in developing countries. Unlike toxigenic strains, which carry the tox gene encoding the diphtheria exotoxin, non-toxigenic strains lack this gene [3]. Although the precise mechanisms of pathogenicity remain unclear, proposed virulence factors include colonisation, adhesion, and epithelial internalisation [4]. Experimental models also suggest that inflammatory responses, particularly via IL-1β and IL-6, contribute to tissue damage and invasive manifestations such as endocarditis, osteomyelitis, bacteraemia, major vascular complications, and septic arthritis [5-7].

In the United Kingdom, national surveillance data demonstrate that reports of non-toxigenic strains increased during the 1990s, peaked in 2000, and subsequently stabilised at 30 to 60 cases annually up to 2022 [7]. In 2024, while only two non-toxigenic infections were formally notified, four cases of infective endocarditis caused by non-toxigenic *C. diphtheriae* were reported, predominantly among vulnerable individuals with risk factors such as substance use, homelessness, or unstable housing [8]. While established guidelines exist for managing toxigenic* C. diphtheriae*, treatment strategies for non-toxigenic strains remain less clearly defined.

## Case presentation

A 19-year-old man presented with a four-day history of feeling generally unwell. His symptoms included lethargy, fever, headache, nausea, and one episode of vomiting. He also complained of new pain and swelling over his left elbow, which started two days ago, and a significantly reduced range of motion. Otherwise, there were no respiratory, genitourinary, or neurological symptoms. He reported no preceding trauma, overuse, or injection drug use. He works as a kitchen porter in a restaurant, does not smoke, and drinks alcohol only occasionally. He denied any sick contacts but reported recent travel to London two weeks earlier. He also reported having unprotected sexual intercourse three months prior to presentation and a history of incomplete immunization during infancy due to side effects that he could not recall.

On examination, the patient was toxic-looking and appeared unwell. While he was pyrexic (38.2°C) and tachycardic (pulse rate 110 beats per minute), the remaining vital signs were unremarkable. Examination of the left elbow revealed mild swelling and tenderness, with a reduced range of motion due to pain. No erythema or warmth was noted over the elbow joint. He was found to have generalised myalgia and arthralgia in the shoulder and knee joints; however, all the joints appeared clinically normal, with no signs of synovitis, warmth, or septic arthritis. Examination of the chest, abdomen, and neurological system was essentially normal. No skin rashes were found.

**Figure 1:**
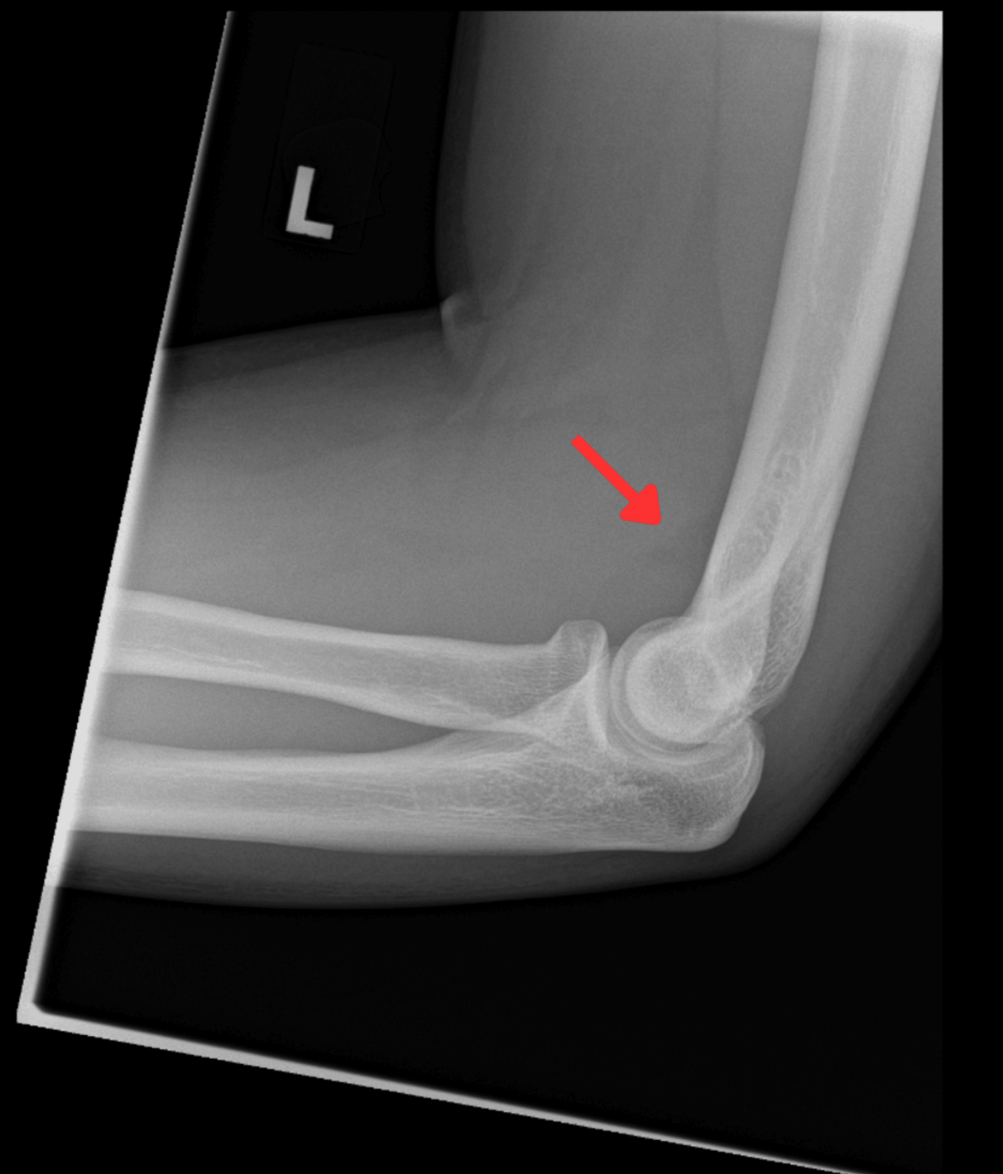
X-ray lateral view of the patient's left elbow Red arrow: Raised anterior fat pad indicating effusion of the left elbow

**Figure 2:**
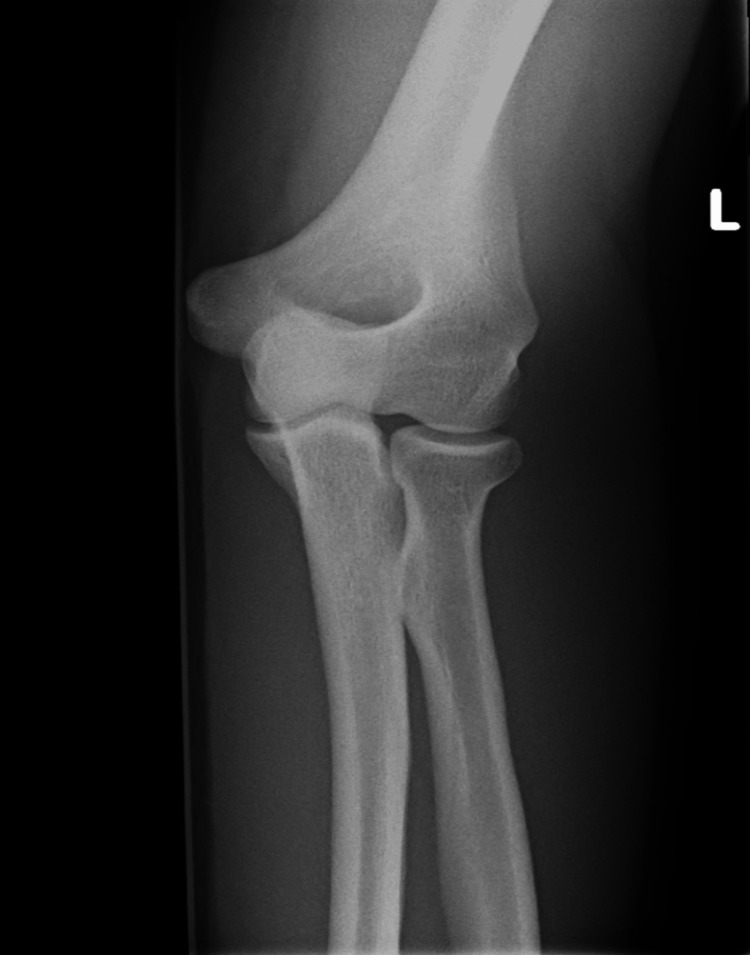
X-ray anteroposterior view of patient's left elbow Anteroposterior view of the left elbow is relatively normal with no features of osteomyelitis or bony abnormalities.

On admission, blood tests revealed an elevated C-reactive protein level of 220 mg/L and a neutrophil count of 7.6 × 10⁹/L; however, the WBC count and lactate levels were normal (Table 1). Tests for blood-borne viruses and sexually transmitted infections, including hepatitis B, hepatitis C, HIV, syphilis, chlamydia, and gonorrhoea, were all negative. An X-ray of the left elbow revealed joint effusion without osteomyelitis or bony abnormalities (Figures 1-2). Left elbow aspiration was done on day two of admission, which revealed purulent synovial fluid, and was sent for culture. Culture of synovial fluid, along with blood culture taken on admission, later grew *C. diphtheriae*. Both culture results were confirmed by the Respiratory and Vaccine Preventable Bacteria Reference Unit (RVPBRU) of the UK Health Security Agency at Colindale as a non-toxigenic strain. No infection source was identified, as throat and nasal swabs for *C. diphtheriae* were negative.

**Table 1:** Patient's laboratory result from day one to day 12 CRP: C-reactive protein

Parameter	Day 1	Day 2	Day 5	Day 10	Day 12	Reference range
WBC count (x10^9^/L)	9.1	12.2	16.0	16.3	14.9	4.0 – 10.0
Neutrophils (x10^9^/L)	7.6	9.9	12.3	13.6	12.3	2.0 – 7.0
Lymphocytes (x10^9^/L)	0.5	1.1	1.9	1.6	1.3	1.1 – 5.0
CRP (mg/L)	220	194	172	203	189	0 – 10
Lactate (mmol/L)	1.7	-	-	-	-	0.6 – 2.2

The initial provisional diagnosis was reactive arthritis with meningitis considered as a differential, and the patient was therefore started on empirical intravenous ceftriaxone and amoxicillin. When *C. diphtheriae* was identified on culture, antimicrobial therapy was switched to intravenous clindamycin, intravenous benzylpenicillin, and oral clarithromycin. Due to the patient being clinically unwell with *C. diphtheriae* bacteraemia, there was concern about diphtheria toxaemia. The patient was subsequently transferred to the high-dependency unit for close monitoring and administration of diphtheria antitoxin, during which no complications, such as anaphylaxis, were noted. He was also given oral linezolid for three days in the high-dependency unit.

Antimicrobial therapy was revised again when the sensitivity report became available (Table 2). The patient was treated with intravenous benzylpenicillin and intravenous clindamycin. He was transferred back to ward-level care on day 11 of admission and showed significant clinical improvement. Intravenous clindamycin was later switched to oral therapy.

**Table 2:** Antibiogram by RVPBRU of UK Health Security Agency at Colindale reported on day 10 RVPBRU: Respiratory and Vaccine Preventable Bacteria Reference Unit; MIC: Minimum inhibitory concentration; S: Susceptible; I: Intermediate; R: Resistant; X: No European Committee on Antimicrobial Susceptibility Testing (EUCAST) clinical breakpoints observed, therefore, formal categorising of the susceptibility of the organism is not possible

Antibiotics	MIC (mg/L)	S/I/R	Breakpoint (mg/L)
Cefotaxime	1	I	0.001 & 2
Penicillin	0.125	I	0.001 & 1
Co-trimoxazole	>32	X	
Vancomycin	0.5	X	
Azithromycin	0.125	X	
Clarithromycin	0.064	X	
Clindamycin	0.5	S	0.5
Erythromycin	0.032	S	0.064
Linezolid	0.5	S	2
Ciprofloxacin	0.064	I	0.001 & 0.5
Doxycycline	0.125	S	0.5
Rifampicin	0.004	S	0.064

The patient’s left elbow swelling had markedly improved, with restored range of motion and significant pain reduction. Arthralgia and myalgia affecting other joints resolved spontaneously, and no residual symptoms were noted. On day 14, he was discharged with oral clindamycin and scheduled for follow-up to monitor joint recovery and symptom resolution. Public health authorities were informed, and close contacts were vaccinated prophylactically.

## Discussion

Literature review of similar cases

A review of the literature reveals that isolated septic arthritis caused by *C. diphtheriae* is an exceptionally rare occurrence, with only three cases reported prior to the current one. This contrasts with septic arthritis secondary to *C. diphtheriae* endocarditis [6,7,9]. The earliest case of septic arthritis caused by *C. diphtheriae* was described by Guran et al. in France in 1979, which involved a two-year-old boy who had a non-toxigenic strain of *C. diphtheriae* identified in his hip joint and skin lesion on his toe, despite being vaccinated [10]. This is followed by a case of isolated wrist septic arthritis caused by a toxigenic strain of *C. diphtheriae* in a 49-year-old female with chronic alcohol use and liver cirrhosis, who was managed with ofloxacin and recovered without long-term complications in 1993 [11]. In the same year, another case of septic arthritis caused by a non-toxigenic strain of* C. diphtheriae* was also reported in a 47-year-old alcoholic with a cirrhotic liver who was not vaccinated [12].

When considering the risk factors for *C. diphtheriae* systemic infections, several factors are commonly implicated in the literature. These include intravenous drug use, low socioeconomic status, poor hygiene, alcoholism, and homelessness [9,13]. Given these risk factors, the primary sites of infection for *C. diphtheriae* were typically the skin or upper respiratory tract. Skin lesions, such as cutaneous ulcers, bullous pemphigoid, scabies, and open fractures, were the main route of bacterial entry, especially in developing countries, accounting for 38% to 50% of cases [9].

This case is noteworthy due to the absence of typical risk factors commonly associated with *C. diphtheriae* systemic infections. The patient is a 19-year-old with no significant medical history, a rare social drinker, and has not been diagnosed with cirrhosis. He did not present with pharyngeal symptoms, and cultures from both the throat and nasal swabs were negative. The only potential risk factor is his lack of vaccination against *C. diphtheriae* and a recent trip to London, which raises the possibility of an atypical or unidentified source of infection. The absence of an obvious primary site of infection makes this case particularly notable. 

Choice of antibiotics

The United Kingdom Health Security Agency (UKHSA) 2024 guidelines recommend IV benzylpenicillin sodium combined with a macrolide as the first-line antibiotics for severe *C. diphtheriae* infections requiring hospitalisation. In cases of extreme systemic illness, additional agents such as linezolid or vancomycin may be considered. This guidance applies broadly, as the primary difference between toxigenic and non-toxigenic strains lies in the presence of a lysogenic bacteriophage carrying the toxin gene (tox), which enables exotoxin production. However, the underlying antibiotic choice remains similar for both toxigenic and non-toxigenic strains [7].

In this case, the patient was started on IV benzylpenicillin, IV clindamycin, and oral linezolid on the second day after a provisional culture identified *C. diphtheriae*. This regimen aligns with UKHSA recommendations, though a macrolide such as erythromycin, clarithromycin, or azithromycin might have been preferable to clindamycin. Nevertheless, subsequent susceptibility testing confirmed that the organism was sensitive to clindamycin, validating the choice. The patient’s complete recovery of joint mobility and resolution of pain further demonstrates the efficacy of the chosen antibiotic regimen.

Diphtheria antitoxin

Diphtheria antitoxin (DAT) neutralises free-circulating toxins, preventing irreversible tissue damage, but its effectiveness diminishes with delayed administration. The UKHSA advises against using DAT for non-toxigenic *C. diphtheriae* strains or asymptomatic carriers, as these strains do not produce toxins, and the risk of anaphylaxis, though extremely rare, outweighs the benefits in such cases. However, the UKHSA guidance provides only general recommendations for managing non-toxigenic corynebacterial infections, noting that clinical management depends on case presentation and site of disease, and acknowledging the paucity of evidence due to the rarity of such infections [7].

In this case, the patient received DAT before PCR results confirmed the absence of the toxin gene, with no anaphylaxis observed. This contrasts with a 2019 Irish case where DAT was withheld in an invasive infection without toxigenic features, highlighting that while antitoxin was likely unnecessary here, further evidence is needed to refine treatment guidelines [7,14].

## Conclusions

Septic arthritis caused by *C. diphtheriae* is an exceptionally rare entity with limited evidence to inform clinical management. This case underscores the importance of recognizing *C. diphtheriae* as a potential pathogen rather than dismissing it as a contaminant, particularly in cases with atypical or subtle clinical presentations. The absence of conventional risk factors further emphasizes the need for heightened clinical vigilance. Ongoing documentation and analysis of such cases are essential to enhance our understanding and guide future management strategies.
